# Soil carbon emissions and influential factors across various stages of vegetation succession in vegetated concrete

**DOI:** 10.1038/s41598-024-56473-9

**Published:** 2024-03-12

**Authors:** Yakun Xu, Ting Luo, Bin Wu, Zhenyao Xia, Wennian Xu, Jiazhen Gao

**Affiliations:** 1 Hubei Provincial Engineering Research Center of Slope Habitat Construction Technique Using Cement-Based Materials, China Three Gorges University, Yichang, China; 2 College of Civil Engineering & Architecture, China Three Gorges University, Yichang, China; 3 Wuhan Polytechnic, Wuhan, 443000 China

**Keywords:** Ecologically restored slopes, Vegetation concrete, Soil respiration, Soil hydrothermal factors, Soil carbon and nitrogen, Restoration ecology, Civil engineering

## Abstract

After ecological restoration of high and steep slopes in the project disturbed area, soil properties, soil microorganisms, litter types and root types change with the succession of vegetation cover communities. However, the effects of different vegetation successional stages on soil respiration dynamics remain unclear. To elucidate trends and drivers of soil respiration in the context of vegetation succession, we used spatio-temporal alternative applied research. Vegetated concrete-restored slopes (VC) with predominantly herbaceous (GS), shrub (SS), and arborvitae (AS) vegetation were selected, and naturally restored slopes (NS) were used as control. SRS1000 T soil carbon flux measurement system was used to monitor soil respiration rate. The results showed that soil respiration (R_S_) and fractions of all four treatments showed a single-peak curve, with peaks concentrated in July and August. During the succession of vegetation from herbaceous to arborvitae on VC slopes, R_S_ showed a decreasing trend, and GS was significantly higher than AS by 45%; Compared to NS, R_S_ was 29.81% and 21.56% higher in GS and SS successional stages, respectively, and 27.51% lower in AS stage. R_S_ was significantly and positively correlated with nitrate nitrogen (NO_3_^–^-N) and microbial biomass nitrogen (MBN), both of which are important factors in regulating R_S_ under vegetation succession. A bivariate model of soil temperature and water content explains the variability of Rs better. Overall, RS was higher than NS in the transition stage and lower than NS in the equilibrium stage of the vegetation community on VC slopes, and the R_S_ decreases gradually with the vegetation succession of artificial ecological restoration slopes.

## Introduction

Soil carbon (C) constitutes 80% of the carbon stock in terrestrial ecosystems, with soil respiration (Rs) representing the second-largest carbon flux between terrestrial ecosystems and the atmosphere (68–98 Pg C year^–1^)^1,2^. Small fluctuations in Rs can have substantial repercussions on atmospheric CO_2_ concentrations, regional and global carbon balances, and, ultimately, climate change^3^. The construction of hydropower stations, mines, roads, and municipal projects, driven by economic development, has led to the destruction of numerous ecosystems. The loss of soil and vegetation has contributed to elevated atmospheric temperatures, as highlighted by the 2021 IPCC report^4^. Consequently, improving degraded ecosystems and mitigating global warming are challenges and major needs for green and high-quality development in China.

Ecological restoration is an effective measure to increase carbon sinks in terrestrial ecosystems and an important means to alleviate global warming. As such, various technologies have been developed to address this problem^5^. Vegetated concrete ecological restoration (VC), as one of the important ecological restoration techniques for disturbed slopes, has been utilized on more than 20 million m^2^ of land in more than 29 provinces, municipalities and autonomous regions in China. It is characterized by a base material made of cement as a binder, mixed with planting soil, organic materials, eco-conditioner, water, and seeds of herbaceous plants and shrubs^6^. This mixture becomes the medium for incorporating herb and shrub seeds, The organic combination of mechanical stability and ecological function has been achieved^7^. However, several questions remain unanswered: What are the carbon emissions from the transition from herbaceous plants and shrubs to trees? What are the differences in carbon emissions between stabilized and naturally restored vegetation communities on slopes under restoration with VC techniques? What factors influence substrate carbon emissions? Accurately estimating VC carbon emissions is crucial for assessing the climatic impact of VC-restored slopes and developing emission reduction strategies for these systems.

Rs consists of autotrophic (Ra) and heterotrophic (Rh) respiration^8^. Ra emanates from plant roots and rhizospheric microorganisms and is closely tied to vegetation characteristics and root activity^9^. In contrast, Rh results from the decomposition of soil organic matter and litter and is linked to substrate availability and microbial activity^10^. Soil temperature and moisture are the primary environmental factors limiting Rs, as they influence soil microbial activity and substrate availability^11,12^. Moreover, interactions between different environmental factors can enhance or counteract their effects on soil C^13^. For instance, research indicates that in arid ecosystems, Rs is constrained by soil moisture, which weakens or abolishes correlations between Rs and temperature^14^. Furthermore in hot and arid conditions Rs exhibits negative temperature sensitivity, with Rs decreasing as temperature rises. This is attributed to further increases in temperature diminishing soil enzyme activity and water availability^15,16^. In another study, Rs initially increased before declining with rising temperature. Thus, temperature and soil moisture directly and indirectly impact Rs^17^. The effects of hydrothermal factors on Rh and Ra in slopes undergoing ecological restoration, especially in VC-restored slopes, remain inadequately studied^18,19^. Therefore, comprehending the influence of hydrothermal factors on VC carbon emissions will furnish a theoretical foundation for the development of VC-based ecological restoration techniques, which are valuable for carbon sequestration in substrate materials and conservation measures.

During ecological restoration employing VC, the choice of herbaceous or shrub seeds depends on soil type and slope gradient. This choice can have substantial implications for the soil microenvironment and root activity, which modulate Ra and Rh. Alterations in vegetation lead to nonlinear changes in soil properties, such as pH, nutrient availability, and its biological parameters, thereby profoundly affecting soil carbon stocks^20^. On the one hand, changes in vegetation and soil nutrients inevitably affect water availability, consequently altering the subsurface soil microbial communities and rate of soil organic matter decomposition^21^. On the other hand, the substrate resources C, N and P in the soil do not fully satisfy the growth requirements of microorganisms, thus creating an imbalance between substrate and microorganisms. This imbalance tends to affect the growth and metabolism of microorganisms and thus the carbon pool in the soil.

To this end, we conducted a year-long experiment in southern China to measure the Rs of naturally restored slopes and VC-restored slopes with various types of vegetation. Additionally, we explored the effects of different factors and vegetation types on Rs. The objectives of this study were to (a) determine monthly changes in VC substrate Rs rates and its composition under different vegetation types and (b) characterizing the response of Rs to hydrothermal conditions, nutrient and litter inputs.

## Materials and methods

### Study area overview

**Figure1:**
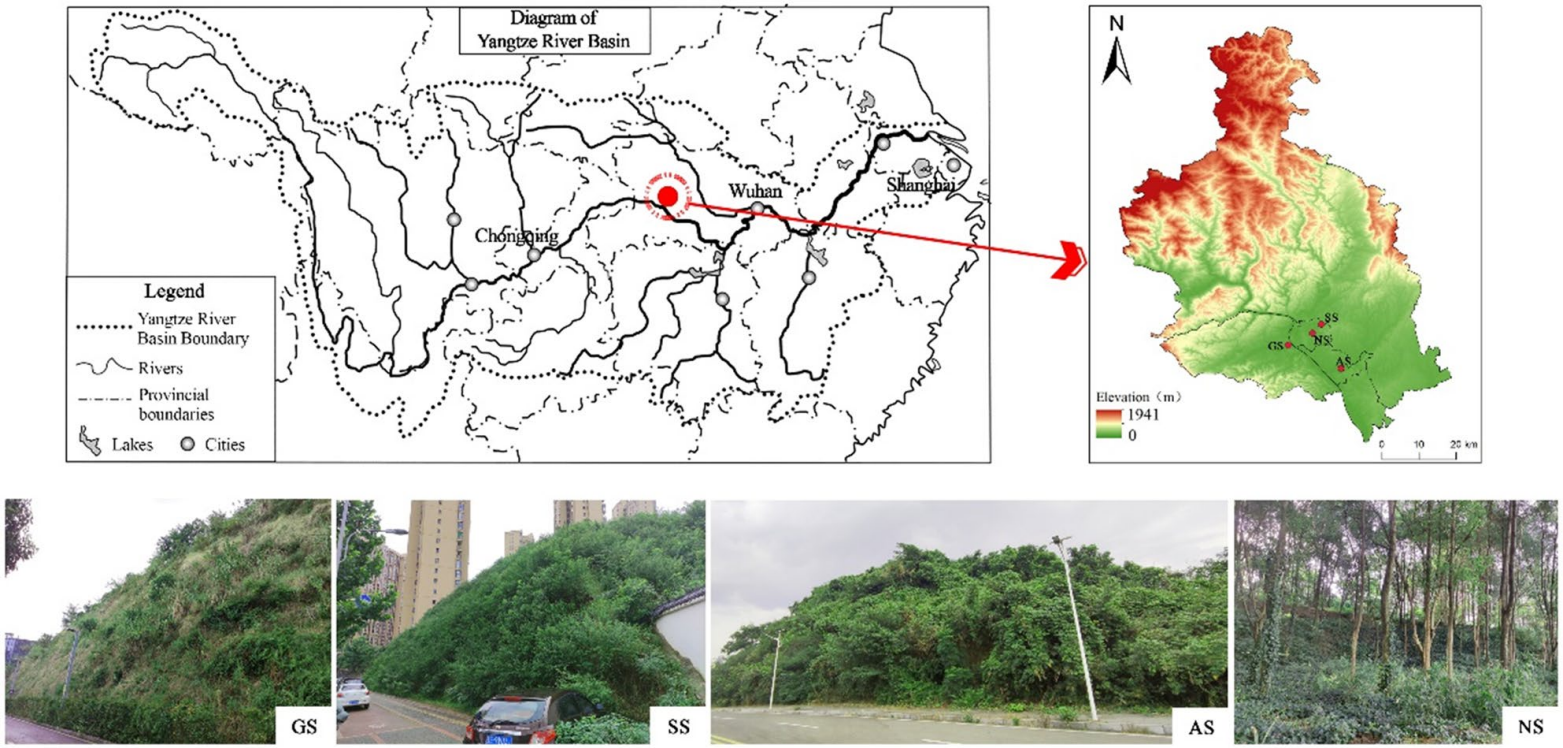
Location of the study area.

The experiment was conducted in an urban area of Yichang City, situated between 29° 56′–31° 34′ N latitude and 110° 15′–112°04′ E longitude, within Hubei Province, China. This study area is characterized by a subtropical monsoon humid climate, with elevation ranging from 63 to 75 m, annual average temperature between 13 and 18 °C, an extensive frost-free period spanning 280 days, and mean annual precipitation level of 992–1404 mm. The area receives abundant rainfall, primarily in late spring and early summer. Soil sampling was conducted in the area by selecting natural restoration slope sample plots and vegetated concrete technology sample plots at different stages of vegetation restoration. The selection of sample plots follows the following principles: ① Typicality of vegetation restoration stages. Using the spatial and temporal substitution method, typical sample plots representing different vegetation restoration measures were selected, including four sample plots of vegetated concrete-restored slopes and naturally restored slopes (NS) with herbaceous (GS), shrub (SS), and arborvitae (AS) vegetation (Fig. 1), each with similar slope orientation (Table 1). ② Similarity of vegetated concrete restoration technique substrate proportioning and spreading seed bank. According to the USDA Soil Taxonomy System, the initial allotment soils of the sample sites were all sandy loam.

**Table 1:** Characteristics of sample areas during various stages of the restoration process.

Sample plot	Longitude and latitude	Repair period (year)	Arboreal coverage (%)	Shrub coverage (%)	Herbaceous coverage (%)	Aspect	Slope (°)	Dominant species
GS	30° 69′ 79.35″ N, 111° 26′ 13.27″ E	2	0	14.7	85.3	ES32°	77	*Digitaria sanguinalis* + *Artemisia argyi* + *Bidens pilosa* + *Festuca elata*
SS	30° 74′ 45.67″ N, 111° 33′ 99.95″ E	4	0	67	18	ES39°	68	*Magnolia multiflora* + *Robinia pseudoacacia*–*Festuca elata*
AS	30° 63′ 94.97″ N, 111° 38′ 79.85″ E	8	58.6	7.3	4.1	ES28°	74	*Broussonetia papyrifera* + *Rhus chinensis*–*Nephrolepis cordifolia*
NS	30° 72′ 52.78″ N, 111° 31′ 72.93″ E	15	32.9	28.7	24.1	ES28°	43	*Chamaecyparis* + *Vitex negundo*–*Artemisia argyi*

### Experimental design and sample processing

Three 10 × 10 m^2^ sample plots were randomly selected in the GS and SS areas, while three 5 × 5 m^2^ sample plots were designated for the AS and NS areas to facilitate long-term observations. In mid-August 2020, Rs measurements were collected in each sample plot using Rs rings. These rings, with an inner diameter of 20 cm and height of 7 cm, had 2 mm holes in the lower part to allow water circulation and plant root penetration. Random placement of these rings on the upper, middle, and lower slopes of each sample area ensured comprehensive data collection. To observe total Rs, 40 × 40 cm^2^ squares were positioned approximately 1 m from each measurement point. For root system partitioning, trenches were dug to depths of 8–10 cm, extending up to the bedrock or below the rhizosphere. Partitions were inserted around the trench to isolate the roots. Meanwhile, all living plants in the squares were removed, Rs rings were buried, and clingfilm was used to halt root development, thereby facilitating Rh determination. Soil was sampled monthly over a year, spanning from January 2022 to December 2022. At each sample plot, soil samples were collected at five locations following an S-type sampling trajectory, and these samples were combined and homogenized. The combined sample was then divided into two portions: one was air-dried and sieved for the measurement of soil physical and chemical properties, whereas the other was stored at 4 °C for soil microbial biomass determination. Litter was collected monthly from 1 × 1 m^2^ areas at the corners and center of each sample plot. The collected litter was dried at 80 °C and used to calculate the litter biomass for each sample plot.

### Soil respiration

Rs was measured using an SRS1000 T soil carbon flux measurement system. Measurements were collected for 2 min at each respiration ring, and all surface vegetation and litter were removed from the respiration ring 24 h prior to measurement^22^. Rs was measured four times each month, from January to December 2022, using a soil respirometer on sunny and windless days. Measurements were collected between 09:00 and 11:00 am. Ra was calculated as the difference between Rs and Rh.

### Environmental factors

The SRS1000 T soil carbon flux measurement system, connected to a LCI thermocouple temperature sensor and EC-5 soil moisture sensor, was used to measure soil temperature and moisture in the 0–5-cm soil layer. Soil chemistry indicators were referenced from *Agricultural Chemical Analysis of Soil* by Bao Shidan. Soil organic carbon (SOC) content was determined using the *potassium dichromate oxidation*-*external heating* method. Soil dissolved organic carbon (DOC) was evaluated using 0.5 mol/L potassium sulfate (with a 2:1 water-to-soil ratio). Soil total carbon (TC) content was determined using a total organic carbon analyzer (MultiN/C2 100, Germany). Soil total phosphorus (TP), total nitrogen (TN), and inorganic nitrogen (NH_4_^+^-N and NO_3_^–^-N) content were determined using a SKALAR San +  + flow analyzer (The Netherlands). Soil microbial biomass carbon (MBC) and microbial biomass nitrogen (MBN) were determined via chloroform fumigation and leaching.

### Statistical data analysis

To assess whether the Rs rate and soil nutrients in different microhabitats within each sample area were significantly different *(P* < 0.05), one-way analysis of variance (one-way ANOVA) was performed using SPSS 21.0 (SPSS Inc., Chicago, IL, USA). Regression modeling between Rs, Rh, Ra and soil temperature and water content. Pearson correlation and principal component analyses were performed between Rs and the soil nutrients at each sample site, and the resulting graphs were plotted using Origin 2021 (Origin Lab., Northampton, MA, USA).

## Results and analysis

### Hydrothermal dynamics of slopes at different stages of vegetation succession

**Figure 2:**
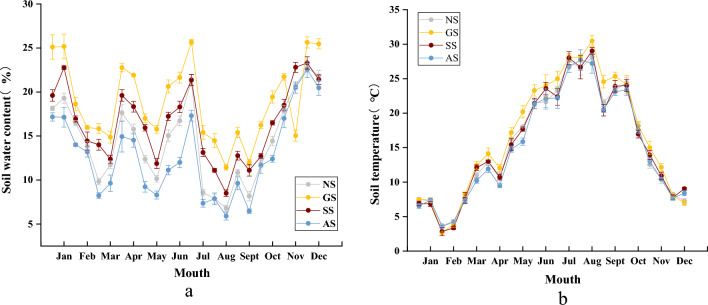
Soil hydrothermal dynamics of slopes in different stages of vegetation succession.

Figure 2a illustrates the fluctuation in soil moisture, exhibiting a multi-peak pattern and reaching its minimum value during the summer months. Subsequently, a rapid increase in soil moisture was observed, coinciding with the onset of rainfall following the high-temperature summer period. Notably, the GS consistently maintained higher soil moisture levels compared to the SS, AS, and NS, particularly during winter and summer. The AS experienced a notable reduction in soil moisture during the high-temperature period, attributed to the lower vegetation coverage on this slope.

In terms of soil temperature, an evident unimodal pattern was observed (Fig. 2b), with temperatures increasing from February to August. The GS consistently exhibited higher temperatures, culminating in a peak value of 30.46 °C in July–August. During the same period, the temperature on the AS was 2.76 °C cooler, registering at 27.7 °C. Subsequently, soil temperature decreased from October to February, with the GS recording the lowest temperatures, and February being the month with the lowest soil temperature. The difference in soil temperatures between the AS and GS was 0.9 °C.

### Soil properties of slopes at different stages of vegetation succession

Significant variations were observed among microhabitats representing the transition from natural vegetation to herbaceous plants, shrubs, and arboreal vegetation in terms of their soil physicochemical properties (Table 2). During the succession from herbaceous to arboreal in VC slopes, pH, SOC, and TN were significantly reduced by 8.15%, 24.88%, and 25% (*P* < 0.05). pH and SOC were significantly higher in VC slopes than in NS (*P* < 0.05), and TN was significantly lower than in NS (*P* < 0.05). The amount of litter fall on VC slopes decreased significantly with succession and was significantly different between GS and AS (*P* < 0.05), and NS was between GS and SS, and both were significantly different (*P* < 0.05). DOC and TC contents tended to decrease and then increase in GS, SS and AS, and AS was significantly higher than NS (*P* < 0.05). For TP content, the difference between groups was not significant (P > 0.05) for VC side slopes, but VC was significantly higher than NS (*P* < 0.05). The size order of NH_4_^+^-N, NO_3_^-^-N, MBC and MBN in VC slopes was SS > GS > AS, in which the differences between NH_4_^+^-N and NO_3_^–^-N were significant in each group, and the MBC and MBN of SS were significantly higher than those of AS.

**Table 2:** Soil properties of slopes at different stages of vegetation succession.

Indicator	NS	GS	SS	AS
pH	7.34 ± 0.20c	8.49 ± 0.28a	8.76 ± 0.40ab	7.80 ± 0.19bc
Litter fall/g·m^–2^	10.17 ± 1.37b	16.19 ± 1.72a	8.32 ± 0.81c	3.90 ± 0.43d
SOC/g·kg^–1^	9.19 ± 0.43c	12.68 ± 0.86a	9.92 ± 0.84b	9.13 ± 0.55c
DOC/mg·kg^–1^	752.81 ± 49.93b	565.58 ± 31.91c	495.33 ± 32.88d	807.51 ± 23.64a
TC/g·kg^–1^	9.55 ± 0.45d	67.23 ± 5.23b	43.44 ± 3.10c	123.56 ± 6.56a
TN/g·kg^–1^	1.62 ± 0.11a	0.66 ± 0.08b	0.74 ± 0.07b	0.52 ± 0.05c
TP/g·kg^–1^	0.60 ± 0.04b	2.17 ± 0.09a	1.98 ± 0.09a	1.72 ± 0.07a
NH_4_^+^-N/mg·kg^–1^	5.66 ± 0.74c	9.97 ± 2.08a	9.47 ± 1.58a	7.21 ± 1.19b
NO_3_^–^-N/mg·kg^–1^	17.15 ± 4.5b	19.65 ± 3.95a	21.91 ± 4.72a	10.27 ± 2.18c
MBC/g·kg^–1^	1.23 ± 0.09a	0.86 ± 0.06bc	0.78 ± 0.06b	0.48 ± 0.05c
MBN/mg·kg^–1^	8.69 ± 0.75b	11.74 ± 1.53a	7.87 ± 1.57b	5.03 ± 0.63c

Lowercase letters indicate significant differences at the *P* < 0.05 level.

### Monthly soil respiration dynamics of the slopes at different stages of vegetation succession and their relationship with hydrothermal factors

**Figure 3:**
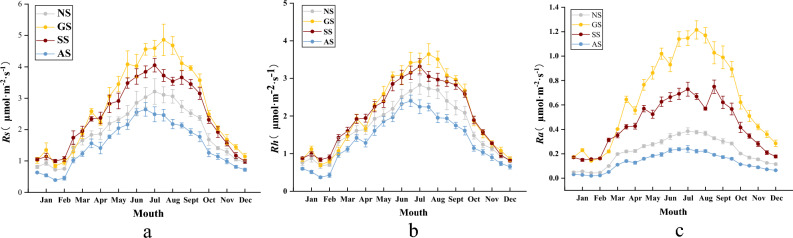
Monthly dynamics of soil respiration in slopes at different stages of vegetation succession. (**a**) Soil respiration (Rs); (**b**) Heterotrophic respiration (Rh); (**c**) Autotrophic respiration (Ra).

**Figure 4:**
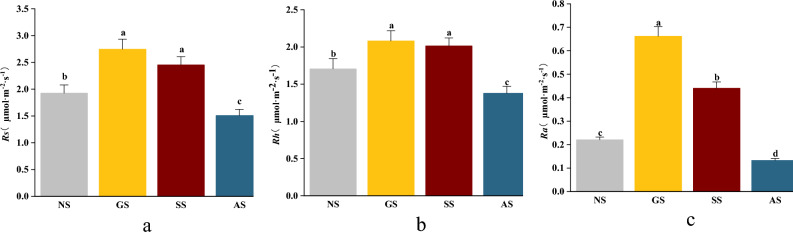
Mean annual soil respiration rates on slopes at different vegetation succession stages. (**a**) Soil respiration (Rs); (**b**) Heterotrophic respiration (Rh); (**c**) Autotrophic respiration (Ra). Lowercase letters denote significant differences at the *P* < 0.05 level.

The monthly patterns of Rs, Rh, and Ra closely mirrored temperature fluctuations, exhibiting a unimodal trend characterized by an initial increase followed by a decrease (Fig. 3). Both Rs and Rh showed a decline from one stage of succession to the next. Concerning the annual mean values of Rs and Rh, no significant difference was observed between GS and SS (*P* > 0.05). However, a noteworthy difference emerged between AS and GS (*P* < 0.05), with Rs and Rh being 44.9% and 33.8% lower in the AS, respectively (Fig. 4). Comparing NS to SS, NS exhibited slightly higher values, surpassing SS by 0.52 μmol CO_2_·m^–2^·s^–1^ for Rs and 0.30 μmol CO_2_·m^–2^·s^–1^ for Rh. Conversely, NS recorded lower values than AS, lagging behind AS by 0.41 μmol CO_2_·m^–2^·s^–1^ for Rs and 0.32 μmol CO_2_·m^–2^·s^–1^ for Rh. Ra displayed significant variation across all sample plots (*P* < 0.05), with AS registering Ra values 79.9%, 69.8%, and 39.7% lower than those of GS, SS, and NS, respectively (Fig. 3).

**Figure 5:**
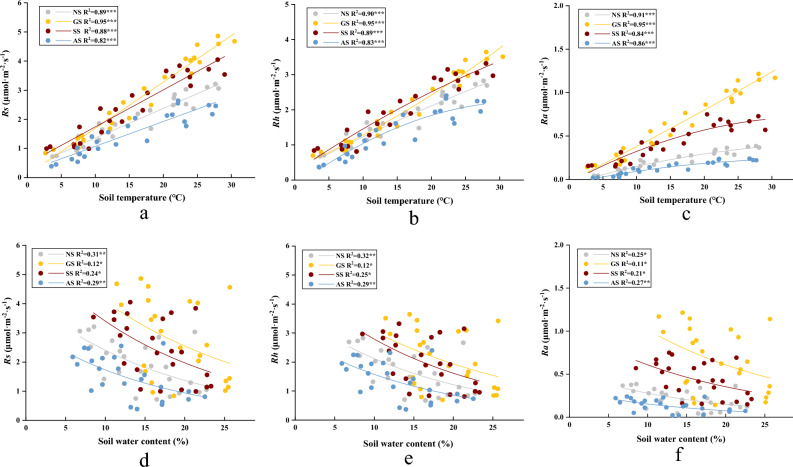
Relationships between monthly soil temperature of slopes at different stages of vegetation succession and soil respiration (Rs, (**a**)), heterotrophic respiration (Rh, (**b**)), and autotrophic respiration (Ra, (**c**)), relationships between water content and Rs (**d**), Rh (**e**), and Ra (**f**). *, **, ***, represent the statistical significance levels: *P* < 0.05, *P* < 0.01, *P* < 0.001.

Soil temperature showed a strong correlation with all Rs components across all stages of succession and sample plots. The coefficients of determination for the effects of soil temperature on Rs, Rh, and Ra were 0.749, 0.822, and 0.412, respectively (Fig. 5). The influence of soil temperature on Rs changes was most pronounced in the GS, followed by SS, NS, and AS. Additionally, there was a negative correlation between Rs rate and soil moisture content (*P* < 0.05), with the coefficient of determination for the effect of soil moisture on Rs rate ranging from 0.12 to 0.31.

From the data presented in Table 3, it is evident that the relationship between the monthly changes of Rs and the multiple linear regression of soil moisture and temperature in various areas is highly significant (*P* < 0.01). The bivariate model incorporating soil moisture and temperature can account for 84% to 96% of the seasonal variations in Rs rates, with the most accurate fit observed in the herbaceous slope (GS).

**Table 3:** Soil properties of slopes at different stages of vegetation succession.

Site	Regression equation	R^2^	*P* value
NS	$Rs=0.991T-0.006W+0.478$	0.91	6.2280e–12***
GS	$Rs=0.162T+0.015W-0.262$	0.96	9.2734e–16***
SS	$Rs=0.125T-0.005W+0.596$	0.89	7.1217e–11***
AS	$Rs=0.082T-0.006W+0.348$	0.84	5.9496e–9***

*Rs* soil respiration, *T* soil temperature, *W* soil water content.

***, represent the statistical significance levels: *P* < 0.001.

### Correlation and principal component analyses of soil respiration and soil properties

**Figure 6:**
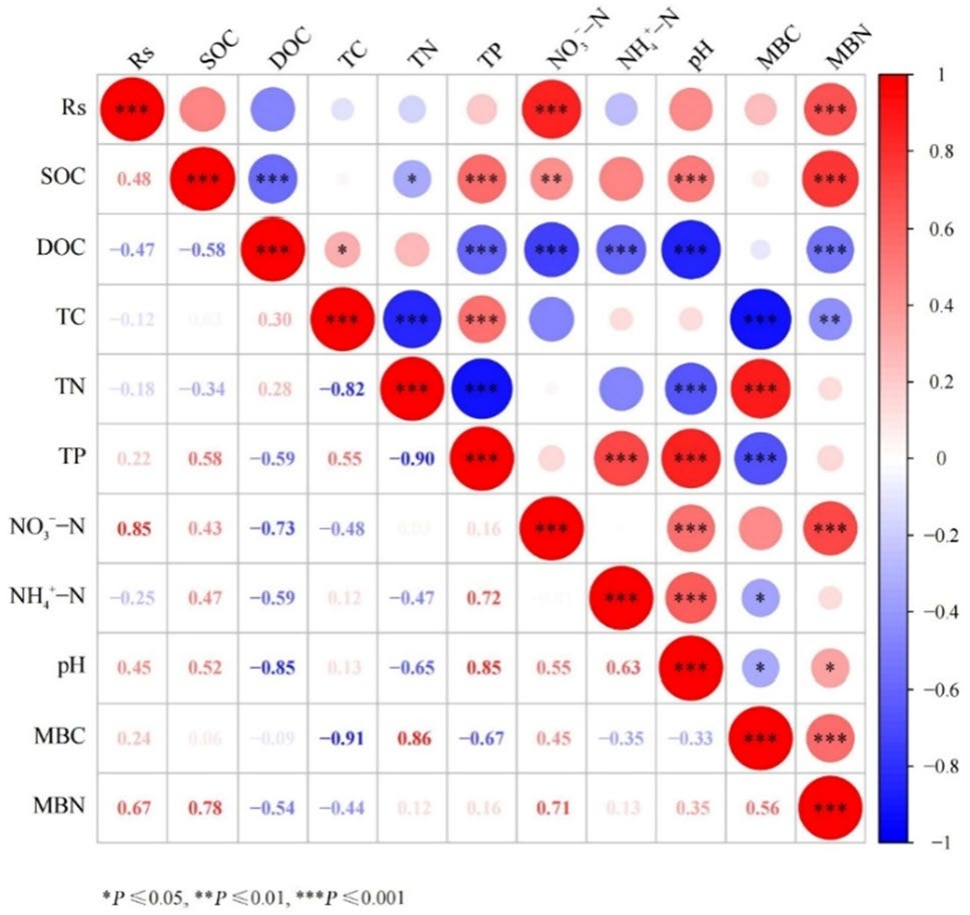
Heatmap illustrating the correlations between Rs and soil environmental factors across slopes at various stages of vegetation succession.

In Fig. 6, Rs evidently exhibits a highly significant positive correlation (*P* < 0.01) with NO_3_^–^-N and MBN, while showing an nonsignificant positive correlation with SOC, pH, and MBC. No significant positive correlation was observed between Rs rate and other nutrients. Notably, SOC displayed a highly significant positive correlation with TP, pH, NO_3_^–^-N, and MBN (*P* < 0.01), and a highly significant negative correlation with DOC (*P* < 0.01). MBC showed a highly significant positive correlation with TN (*P* < 0.01) and a significant negative correlation with TC, TP, pH, and NO_3_^–^-N (*P* < 0.01 and *P* < 0.05).

**Figure 7:**
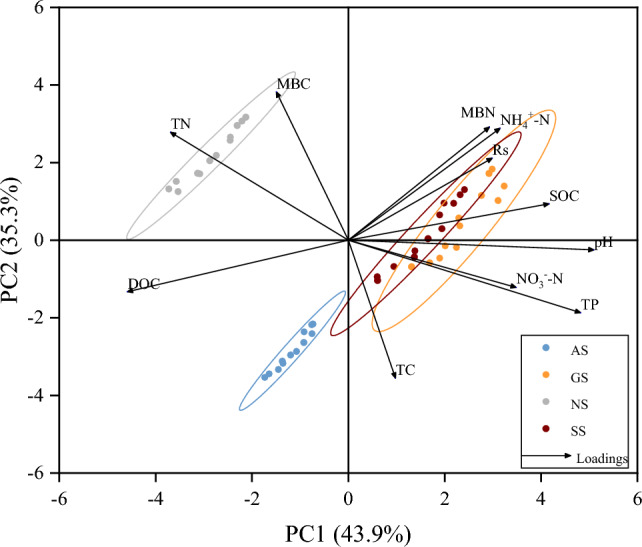
Principal component analysis of the relationship between Rs and soil environmental factors on slopes at different vegetation succession stages.

The principal component analysis results presented in Fig. 7 reveal that the first two principal components (PC1 and PC2) collectively account for 79.2% of the variance. PC1 contributes 43.9% and possesses an eigenvalue of 4.82, whereas PC2 contributes 35.3% with an eigenvalue of 3.88. The directional arrows of NO_3_^–^-N, MBN, SOC, pH, and TP align with that of Rs, indicating a positive correlation with Rs. Additionally, the angles between Rs and TC, TN, MBC, and DOC exceed 90°, suggesting a positive correlation between Rs and the consumption rate of soil organic nutrients and an increase in inorganic nutrient content.

## Discussion

### Effect of vegetation succession stage on slope substrate soil respiration

Ecological restoration serves as a potent tool to enhance terrestrial ecosystem carbon sequestration, a pivotal strategy in mitigating global warming^23^. However, this endeavor brings forth alterations in vegetation cover that impact SOC stability^24^. The intricate interplay between vegetation characteristics, physiological responses, and growth patterns introduces complexity to the study of Rs^25^. Our findings reveal a discernible decrease in monthly values of Rs, Rh, and Ra as the transition from GS to AS unfolds. The decline stems from multiple factors. Changes in soil characteristics due to shifting vegetation impact both microbial communities and soil gas diffusion properties. This observation aligns with the work of Wu et al.^26^.

The influence of vegetation type on Rs is multifaceted. Herbaceous plants, prevalent on high and steep slopes, provide extensive cover. This reduces solar radiation and surface evapotranspiration while augmenting water-use efficiency and microbial activity, notably enhancing Rh^27^. The substrate thickness on steep slopes generally ranges from 8 to 12 cm, compared to 10 cm for VC^28^. Herbaceous plants have shorter root lengths and increased root content compared to AS^29^. The nutrient-rich substrate on GS fosters root activity in herbaceous plants, subsequently elevating Ra (Table 2). As Rh and Ra gradually diminish during community succession on VC slopes, Rs decreases from one stage to the next. The dense and low vertical distribution of plant communities on GS facilitates litter accumulation, particularly during the non-growing season. Drying herbaceous roots and litter, characterized by low lignin and cellulose content, readily decompose^30^. Consequently, GS contribute more carbon to the substrate, expediting soil microbial community development and enhancing Rs^31^. These findings differ from those of Chen^32^, potentially attributable to our focus on slopes with gradients exceeding 65° (Table 1), where litter remains on shrubby and arboreal slopes due to gravity, wind, and rainfall, in contrast to plains where Chen conducted experiments.

Compared with naturally restored slopes, ecologically restored slopes had significantly higher Rs in the early stage of restoration and significantly lower Rs in the late stage of restoration. It is possible that the significant elevation of substrate (SOC) in the soil after inputs in the eco-conditioner favored the increase of respiratory substrate effectiveness of soil microorganisms and the promotion of root respiration in plants, which in turn increased Rh and Ra. Over time, rainfall leached these nutrients from the substrate, resulting in reduced Rs during the later stages of restoration.

### Soil respiration and hydrothermal dynamics in different vegetation succession stages on slopes

In the context of climate change, quantifying how key environmental factors, such as soil moisture and temperature, modulate Rs’s role in the carbon source/sink balance of ecosystems is crucial^33^. The type of vegetation profoundly influences soil microbial activity and respiration by altering topsoil temperature and moisture levels.

During the transition from GS to AS, monthly Rs dynamics align with substrate temperature variations, exhibiting a decline as succession progresses. This pattern is attributed to temperature-induced increases in microbial decomposition of soil organic matter and nitrogen mineralization, relieving nutrient limitations in the slope substrate and stimulating plant nitrogen uptake, thereby elevating Rs. Correlation analysis between Rs and temperature in VC substrate yields significant linear and exponential correlations, consistent with previous research^34,35^. GS dampen temperature fluctuations by reducing the diurnal temperature difference through vegetation^36^, which may account for the higher fit of Rs to temperature for GS than for AS.

Soil moisture plays a vital role in stimulating soil microbe respiration and autotrophic respiration by enhancing microbial substrate availability, microbial biomass, and plant growth^37^. Moisture-induced changes in soil physical properties also impact CO_2_ emissions. In this study, Rs exhibits a negative correlation with soil moisture, suggesting that elevated moisture levels inhibit Rs. This inhibition may arise from increased soil particle adhesion, diminishing soil permeability and oxygen availability^38^. Elevated soil moisture content could also reduce rhizospheric and microbial activities, negatively affecting Rs^39^. The two-factor interaction of soil temperature and soil water content may better explain the variability of Rs, i.e. vegetation succession dominates the effects of soil temperature and soil water content on Rs^40^.

### Impact of soil physicochemical properties on soil respiration across vegetation succession stages on slopes

Soil nutrient enrichment follows numerous pathways, encompassing hydrological, rhizospheric, vegetation, and animal contributions^41^. In our study, the GS boast nutrient-rich substrates, stimulating microbial metabolism and elevating Rs. Conversely, TP, TN, and inorganic nitrogen content gradually decline over time on SS and AS, contributing to reduced Rs. The lower Rs on these slopes can also be attributed to the high levels of recalcitrant lignin in their litter^42^. Furthermore, arboreal plant leaves generally exhibit lower C/N ratios compared to herbaceous plants, which may enhance soil microbial biomass and activity (Rs and mineralization). This interaction leads to the integration of litter with rhizospheric biomass, limiting the accumulation of C and N in near-surface soils^43^. The low nutrient content on NS serves as the primary reason for their lower Rs compared to VC-restored slopes.

We found that vegetation succession modulates the degree of influence of slope ecosystems on Rs, and this influence modulation is mainly governed by soil physicochemical properties, especially soil NO_3_^–^-N and MBN. Because NO_3_^–^-N promotes microbial-mediated Rs, nitrogen transformations in the soil such as nitrification increase soil acidification, coupled with the fact that vegetated concrete is an alkaline substrate, carbonate-containing soils neutralize acidity and release CO_2_^44^, which manifests itself in the early stages of succession as the highest levels of NO_3_^–^-N. At the same time, the positive interaction between substrate structure and ecological amendment decay in the early stage of succession increased the effective soil nitrogen content, thus enhancing microbial fixation of nitrogen and improving nitrogen uptake and utilization by fine roots. Rs is reduced as soil temperature and litter inputs decrease, inhibiting microbial activity and thus reducing microbial species richness and diversity^45^. In addition, Rs was also positively correlated with SOC, as shown by the gradual decrease of SOC during vegetation succession, followed by the decrease of Rs. The main reason was the rapid decomposition of organic matter due to the increase in soil temperature and substrate quality, consistent with the results of Liu et al.^46^. In addition, there is also partly originated from microbial-mediated heterotrophic respiration. In this study, Rs was found to be negatively correlated with DOC, while soil heterotrophic respiration was mainly associated with fungi or with fungus-associated bacteria^20^, also noted that the α-diversity of bacteria is driven by DOC and the β-diversity is mainly driven by water content. The evolution of herbaceous to arboreal populations may cause a shift from fungal to bacterial populations, thereby reducing Rs.

Overall, VC ecological restoration techniques reduced slope Rs in the later stages of vegetation succession, the supply of substrates (e.g. SOC) such as VC substrates with the addition of eco-conditioner significantly increases the level of soil microbial metabolism, which further confirms that ecological restoration is an important measure to reduce "carbon sources" in ecosystems. Therefore, the application of VC ecological restoration technology in the ecological restoration process of slopes can reduce the carbon emissions in the disturbed area of the project, which is an effective method to help achieve carbon neutrality.

## Conclusions

In this study, we found that monthly changes in R_S_ and its components were consistent with changes in soil temperature, showing a clear single-peak curve, with peaks occurring in July and August. VC restoration techniques showed a decreasing trend in soil respiration rates during vegetation succession, with a 45% reduction in AS compared to GS and a 27.51% reduction in AS compared to NS. Soil temperature and moisture content are the main abiotic factors affecting soil respiration. R_S_ was significantly and positively correlated with NO_3_^–^-N and MBN, both of which are important factors in regulating R_S_ under vegetation succession. Soil physicochemical properties explained 79.2% of the soil respiration rate. Overall, succession of vegetation cover types on high and steep slopes reduces Rs, and soil temperature and soil moisture content play an important role in regulating soil respiration. VC slopes have lower Rs than NS slopes in the later stages of ecological restoration, and the application of VC technology can help to reduce soil carbon emissions in project-disturbed areas.

## Data Availability

Additional Supporting Information may be found online at: https://www.scidb.cn/s/zuqMfm. The intended repository where data will be permanently archived if the paper is accepted for publication.
